# The association of *POLR2E* rs3787016 polymorphism and cancer risk: a Chinese case–control study and meta-analysis

**DOI:** 10.1042/BSR20180853

**Published:** 2018-11-16

**Authors:** Bifeng Chen, Shang Wang, Guangxin Ma, Jin Han, Jingli Zhang, Xiuli Gu, Xianhong Feng

**Affiliations:** 1 Department of Biological Science and Technology, School of Chemistry, Chemical Engineering and Life Sciences, Wuhan University of Technology, Wuhan, China; 2 Center of Reproductive Medicine, Tongji Medical College, Huazhong University of Science and Technology, Wuhan, China; 3 Department of Reproductive Genetics, Wuhan Tongji Reproductive Medicine Hospital, Wuhan, China; 4 Clinical Laboratory, Wuhan Xinzhou District People’s Hospital, Wuhan, China

**Keywords:** long non-coding RNA, liver cancer, lung cancer, meta-analysis, POLR2E, rs3787016

## Abstract

How single nucleotide polymorphisms in long non-coding RNAs are involved in cancer susceptibility remains poorly understood. We hypothesized that polymerase II polypeptide E (*POLR2E*) rs3787016 polymorphism, identified in a genome-wide association study of prostate cancer, might be a common genetic risk factor for cancer risk. To address this issue, we here conducted a case–control study to investigate the association of *POLR2E* rs3787016 polymorphism with risk of liver and lung cancer (including 800 normal controls, 480 liver cancer patients, and 550 lung cancer patients), followed by a meta-analysis. The genotyping was performed by polymerase chain reaction-restriction fragment length polymorphism and confirmed by sequencing. Although no significant association was found for rs3787016 with risk of liver or lung cancer, the further stratified analysis identified that rs3787016 contributed to liver cancer risk particularly for over than 60 years individuals who drink. Moreover, the meta-analysis demonstrated that rs3787016 was associated with overall cancer risk and prostate cancer risk. Collectively, the *POLR2E* rs3787016 polymorphism may be a valuable biomarker for cancer predisposition.

## Introduction

Liver and lung cancers are commonly diagnosed cancers with high mortality rate in China [1,2]. Although great progress has been made in diagnosis and treatment of cancers over the past decade, the 5-year overall survival rates of lung and liver cancer patients remain low [3]. The major reason is that most patients are diagnosed at advanced stage, with consequently poor prognosis and limited treatment options. Therefore, it is emergent to identify certain inherited genetic variants associated with susceptibility to liver and lung cancer, which would be in favor of making early diagnosis and risk prediction.

Long non-coding RNAs (LncRNAs) are non-protein coding transcripts usually between 200 kb and 1000 kb in length and play important roles in diverse cellular processes, like growth, difference, apoptosis, epigenetic, and gene expression regulation [4]. Aberrant expression of lncRNAs has been identified in many cancer types, including liver and lung cancer, suggesting that lncRNAs might be involved in tumorigenesis and tumor progression [5]. In addition, single nucleotide polymorphism (SNP), which can affect the expression and function of genes, has been reported to be associated with susceptibility to many kinds of human complex diseases including cancer [6].

Rs3787016, which localizes to the fourth intron of RNA polymerase II polypeptide E (*POLR2E*) gene, has been studied by several researchers on its association with cancer risk [7–11]. However, the results remain conflicting rather than conclusive, probably due to the small sample size and different ethnic backgrounds of participants. To date, no study has been conducted to investigate the association between the risk of liver or lung cancer and *POLR2E* rs3787016 polymorphism. In view of this, a case–control study, based on 480 liver cancer patients, 550 lung cancer patients, and 800 normal controls, was conducted to evaluate the association between *POLR2E* rs3787016 and risk of lung and liver cancer in a Chinese population of Hubei province. Besides, we further carried out a meta-analysis, combining results from previous published literature and our case–control study, to clarify the real influence of rs3787016 on cancer risk.

## Materials and methods

### Participants

The participants were consisted of 480 patients with histologically confirmed liver cancer, 550 patients with histologically confirmed lung cancer, and 800 cancer-free controls. The liver and lung cancer patients were volunteers recruited from Hubei Cancer Hospital and Wuhan Xinzhou District People’s Hospital between January 2015 and December 2016, while the normal controls were selected from visitors who came to Wuhan Xinzhou District People’s Hospital for regular physical examinations between September 2014 and December 2016. All subjects were biologically unrelated Han Chinese living in Hubei province. The present study was approved by the Ethical Committees of Wuhan University of Technology and written informed consent for the genetics analysis was obtained from all subjects or their guardians.

### The genotyping of POLR2E rs3787016 polymorphism

Genomic DNA was extracted from venous blood using the TIANamp Blood DNA Kit (DP348, TianGen Biotech, Beijing) according to the manufacturer’s instructions, and stored at −20°C before used. Polymerase chain reaction-restriction fragment length polymorphism (PCR–RFLP) was used to genotype the *POLR2E* rs3787016 polymorphism. The PCR primers were designed by Primer Premier 6.0 (PREMIER Biosoft), and the sequences were: 5′-CATCAACATCACGCAGCACG-3′(forward) and 5′-CCCTGTCCTCCAAGCACTCAT-3′(reverse). The PCR annealing temperature was 60°C. The transition of T > C at rs3787016 polymorphism produces a *NLaIII* restriction site. Therefore, the 147 bp fragment of PCR product was then digested with *NLaIII* (Takara Biotechnology Co. Ltd, Dalian, China) overnight at 37°C, and the digested DNA fragmentations were evaluated by 2.5% agarose gel electrophoresis. The rs3787016 C allele results in two bands (127 bp and 20 bp), while the T allele produces one band (147 bp). For quality control, genotyping analysis was repeated twice. Furthermore, 20% randomly selected PCR-amplified DNA samples were examined by DNA sequencing, and the results were 100% concordant.

### Statistical analysis

All statistical analyses were performed by SPSS 15.0 software (SPSS, Chicago, IIIinois). The χ^2^ test was used to compare the differences in age, gender, smoking status, and drinking status between cancer patients and healthy controls. Hardy–Weinberg equilibrium (HWE) for rs3787016 genotype was tested by Pearson χ^2^ test statistics amongst the normal controls. Association between rs3787016 and cancer risk was assessed by unconditional logistic regression analysis with odds ratios (ORs) and 95% confidence intervals (CIs). Six genetic models, including T vs. C (allele model), TT vs. CT (carrier model: T carrier vs. C carrier), TT vs. CC (homozygote model), CT vs. CC (heterozygote model), TT vs. CT + CC (recessive model) and TT + CT vs. CC (dominant model) were used. The criterion of statistical significance was set at *P*<0.05, and Bonferroni correction for multiple testing was applied [12].

### Meta-analysis

**Figure 1:**
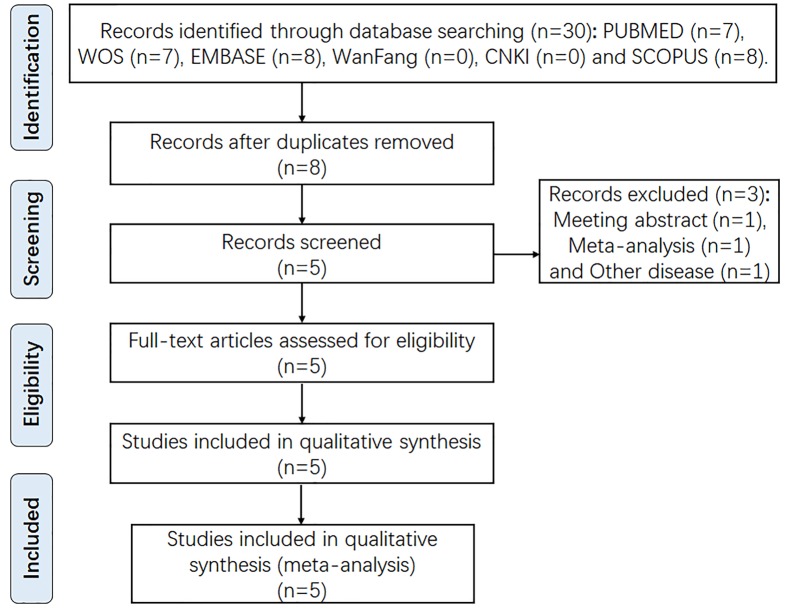
Flow diagram of the literature review process for *POLR2E* rs3787016 polymorphism and cancer risk

We comprehensively searched the EMBASE, PubMed, ISI Web of Science, China National Knowledge Infrastructure, and WanFang databases updated to April 2018 to identify the eligible studies. The search details were shown in Supplementary Table S1. Flowchart of the search strategy and article selection for meta-analysis was demonstrated in Figure 1. References listed in retrieved articles were also checked for missing information. Moreover, eligible studies were included while they met the following inclusion criteria: (1) studies on humans; (2) investigation of the POLR2E rs3787016 polymorphism and cancer risk; (3) case–control study design; (4) valid data were accessible to estimate the OR and its 95% CI; (5) HWE equilibrium should be established in control groups. Finally, five relevant articles were retrieved [7–11]. The Newcastle-Ottawa Scale (NOS) was used to assess the quality of included studies [13]. The meta-analysis was conducted by Review Manager 5.3 (Cochrane Collaboration). Different ethnicity descents were categorized as Asian and Caucasian. Heterogeneity was evaluated with the χ^2^ test and the inconsistency index (*I*^2^), and heterogeneity was considered significant when *P*<0.1 was consistent with possible substantial heterogeneity. If *P*<0.1, random-effects model was conducted to calculate the combined OR [14], otherwise, fixed-effect model we used [15]. The significance of combined ORs of the six genetic models (allele, carrier, homozygote, heterozygote, recessive, and dominant) was determined by the *Z* test. Further, sensitivity analysis was also tested by removing one study at a time, to evaluate the effect of removal and effect of size of each study on the homogeneity of the whole.

**Table 1:** Characteristics of liver cancer patients, lung cancer patients, and normal controls

Variables	Liver cancer patients (*n*=480)	Lung cancer patients (*n*=550)	Normal controls (*n*=800)	*P* value^2^	*P* value^3^
**Age (years)**					
≤60	280 (58.3%)^1^	306 (55.6%)	434 (54.3%)	0.154	0.615
>60	200 (41.7%)	244 (44.4%)	366 (45.7%)		
**Gender**					
Male	343 (71.5%)	373 (67.9%)	558 (69.7%)	0.517	0.451
Female	137 (28.5%)	177 (32.1%)	242 (30.3%)		
**Smoking status**					
Ever	140 (29.2%)	150 (27.3%)	209 (26.1%)	0.237	0.639
Never	340 (70.8%)	400 (72.7%)	591 (73.9%)		
**Alcohol status**					
Ever	158 (32.9%)	170 (31.0%)	237 (29.6%)	0.217	0.613
Never	322 (67.1%)	380 (69.0%)	563 (70.4%)		

1 Numbers in parentheses, percentage.

2 Age, gender, smoking status, and alcohol status distributions of liver cancer patients and normal controls were compared using two-sided χ^2^ test.

3 Age, gender, smoking status and alcohol status distributions of lung cancer patients and normal controls were compared using two-sided χ^2^ test.

## Results

### Characteristics of participants

Table 1 showed us the main characteristics of participants. No significant differences for the distributions of age, gender, smoking status, and drinking status was identified between liver cancer patients and healthy controls, as well as between lung cancer patients and healthy controls. These results indicated that our case–control study was well matched based on these four variables.

### Association of POLR2E rs3787016 polymorphism with risk of liver and lung cancer

In the present study, rs3787016 was successfully genotyped in a total of 1830 participants. The allele and genotype distributions of rs3787016 and their association with risk of liver and lung cancer were presented in Table 2. The genotype frequencies of rs3787016 in normal controls showed no significant deviation from the HWE (*P*=0.205). As shown in Table 2, the allele and genotype distributions of rs3787016 showed no significant differences between liver or lung cancer patients and normal controls. Further logistic regression analysis under the six genetic models (T vs. C, TT vs. CT, TT vs. CC, CT vs. CC, TT vs. CT + CC, and TT + CT vs. CC) revealed no significant association between *POLR2E* rs3787016 and risk of liver or lung cancer.

**Table 2:** Genotype and allele distributions of *POLR2E* rs3787016 polymorphism and its association with the risk of liver and lung cancer

POLR2E rs3787016	I. Liver cancer patients (*n*=480)	II. Lung cancer patients (*n*=550)	III. Normal controls (*n*=800)	*P* value^2^		Logistic regression [*P*, OR (95% CI)]^3^		
				I vs*.* III	II vs*.* III	Genetic Model	I vs. III	II vs. III
T	576 (60%)^1^	612 (55.6%)	936 (58.5%)	0.455	0.139	T vs. C	0.455, 1.06 (0.90–1.25)	0.139, 0.890 (0.76–1.04)
C	384 (40%)	488 (44.4%)	664 (41.5%)					
TT	188 (39.2%)	181 (32.9%)	286 (35.8%)	0.373	0.346	TT vs*.* CT	0.164, 1.20 (0.93–1.54)	0.515, 0.92 (0.72–1.18)
CT	200 (41.7%)	250 (45.5%)	364 (45.5%)			TT vs*.* CC	0.669, 1.07 (0.78–1.47)	0.145, 0.80 (0.59–1.08)
CC	92 (19.2%)	119 (21.6%)	150 (18.8%)			CT vs*.* CC	0.489, 0. 96 (0.66–1.22)	0.329, 0.87 (0.65–1.16)
						TT vs*.* CT + CC	0.221, 1.16 (0.92–1.46)	0.281, 0.88 (0.70–1.11)
						TT + CT vs*.* CC	0.854, 0.97 (0.73–1.29)	0.192, 0.84 (0.64–1.10)

1 Numbers in parentheses, percentage.

2 The frequencies of allele and genotype in cancer patients and normal controls were compared using two-sided χ^2^ test.

3 The *P* value was calculated using two-sided χ^2^ test. OR (95% CI) was estimated by logistic regression analysis.

### Stratified analysis of the association between rs3787016 polymorphism with risk of liver and lung cancer according to age, gender, smoking status, and alcohol status

Considering the importance of age, gender, smoking, and drinking in liver and lung carcinogenesis [16,17]; thus, we conducted a stratified analysis of rs3787016 according to these four variables. All genotype frequencies of rs3787016 were consistent with the HWE amongst normal controls in each subgroup (*P*>0.05). According to the results in Table 3, it was interestingly to find an increased liver cancer risk for rs3787016 T allele and TT genotype in older participants (T vs*.* C: *P*=0.005, OR = 1.44, 95% CI = 1.12–1.86; TT vs*.* CC: *P*=0.005, OR = 2.22, 95% CI = 1.27–3.89) and ever drinking participants (T vs*.* C: *P*=0.002, OR = 1.58, 95% CI = 1.18-2.12; TT vs*.* CC: *P*=0.003, OR = 2.49, 95% CI = 1.36–4.58) even after Bonferroni correction (*P*<0.008, 0.05/6). These results suggested potential interactions amongst rs3787016, aging, and drinking in the etiology of liver cancer. However, our results revealed no significant association between rs3787016 and lung cancer risk in none of the stratified analysis by age, gender, smoking status, and drinking status.

**Table 3:** Stratification analyses of *POLR2E* rs3787016 genotype and allele according to age, gender, smoking status, and drinking status

Groups	Allele		Genotype				Logistic regression [*P*, OR (95% CI)]^2^					
	T	C	TT	CT	CC	HWE^1^	T vs*.* C	TT vs*.* CT	TT vs*.* CC	CT vs*.* CC	TT vs*.* CT + CC	TT + CT vs*.* CC
**≤60 years**												
Liver cancer patients	310	250	101	108	71		0.176, 0.86 (0.70–1.07)	0.573, 1.10 (0.78–1.56)	0.131, 0.73 (0.49–1.10)	0.043, 0.66 (0.45–0.99)	0.781, 0.96 (0.70–1.31)	0.049, 0.70 (0.49–1.00)
Lung cancer patients	341	271	101	139	66		0.210, 0.88 (0.71–1.08)	0.363, 0.86 (0.62–1.19)	0.255, 0.79 (0.53–1.19)	0.675, 0.92 (0.62–1.36)	0.252, 0.84 (0.61–1.14)	0.414, 0.86 (0.60–1.24)
Normal controls	512	356	161	190	83	0.139						
**>60 years**												
Liver cancer patients	266	134	87	92	21		0.005, 1.44 (1.12–1.86)	0.148, 1.32 (0.91–1.91)	0.005, 2.22 (1.27–3.89)	0.063,1.69 (0.97–2.93)	0.028, 1.48 (1.04–2.11)	0.016, 1.91 (1.13–3.23)
Lung cancer patients	271	217	80	111	53		0.409, 0.91 (0.72–1.14)	0.986, 1.00 (0.69–1.45)	0.363, 0.81 (0.51–1.27)	0.329, 0.81 (0.52–1.24)	0.726, 0.94 (0.67–1.33)	0.299, 0.81 (0.54–1.21)
Normal controls	424	308	125	174	67	0.895						
**Male**												
Liver cancer patients	411	275	134	143	66		0.583, 1.06 (0.87–1.28)	0.255, 1.19 (0.88–1.61)	0.779, 1.06 (0.72–1.54)	0.526, 0.89 (0.61–1.29)	0.331, 1.15 (0.87–1.52)	0.822, 0.96 (0.68–1.35)
Lung cancer patients	416	330	123	170	80		0.225, 0.89 (0.74–1.07)	0.577, 0.92 (0.68–1.24)	0.233, 0.80 (0.55–1.16)	0.436, 0.87 (0.61–1.24)	0.368, 0.88 (0.67–1.16)	0.292, 0.84 (0.61–1.16)
Normal controls	654	462	200	254	104	0.344						
**Female**												
Liver cancer patients	165	109	54	57	26		0.599, 1.08 (0.80–1.47)	0.420, 1.21 (0.76–1.93)	0.726, 1.11 (0.62–2.00)	0.768, 0.92 (0.52–1.63)	0.452, 1.18 (0.77–1.82)	0.994, 1.00 (0.59–1.71)
Lung cancer patients	196	158	58	80	39		0.403, 0.89 (0.67–1.17)	0.737, 0.93 (0.60–1.44)	0.407, 0.80 (0.46–1.37)	0.559, 0.86 (0.51–1.44)	0.556, 0.88 (0.59–1.33)	0.447, 0.83 (0.51–1.34)
Normal controls	282	202	86	110	46	0.596						
**Ever-smoking**												
Liver cancer patients	168	112	55	58	27		0.715, 1.06 (0.78–1.44)	0.452, 1.20 (0.75–1.94)	0.851, 1.06 (0.58–1.93)	0.676, 0.88 (0.49–1.59)	0.520, 1.16 (0.74–1.80)	0.884, 0.96 (0.56–1.66)
Lung cancer patients	168	132	50	68	32		0.485, 0.90 (0.67–1.21)	0.769, 0.93 (0.58–1.50)	0.489, 0.81 (0.45–1.46)	0.634, 0.87 (0.50–1.53)	0.617, 0.89 (0.57–1.39)	0.531, 0.85 (0.50–1.43)
Normal controls	245	173	75	95	39	0.660						
**Never-smoking**												
Liver cancer patients	408	272	133	142	65		0.515, 1.07 (0.88–1.29)	0.242, 1.19 (0.89–1.61)	0.701, 1.07 (0.74–1.57)	0.580, 0.90 (0.62–1.30)	0.299, 1.16 (0.88–1.52)	0.900, 0.98 (0.70–1.36)
Lung cancer patients	444	356	131	182	87		0.191, 0.89 (0.74–1.06)	0.559, 0.92 (0.69–1.22)	0.199, 0.79 (0.56–1.13)	0.394,0.86 (0.62–1.21)	0.338, 0.88 (0.67–1.15)	0.252, 0.83 (0.61–1.14)
Normal controls	691	491	211	269	111	0.311						
**Ever-drinking**												
Liver cancer patients	206	110	68	70	20		0.002, 1.58 (1.18–2.12)	0.151, 1.39 (0.89–2.16)	0.003, 2.49 (1.36–4.58)	0.053, 1.80 (0.99–3.26)	0.021, 1.63 (1.08–2.48)	0.010, 2.09 (1.19–3.64)
Lung cancer patients	181	159	51	79	40		0.781, 0.96 (0.73–1.27)	0.726, 0.92 (0.58–1.46)	0.808, 0.94 (0.54–1.61)	0.953, 1.02 (0.62–1.67)	0.723, 0.93 (0.60–1.42)	0.940, 0.98 (0.62–1.56)
Normal controls	257	217	75	107	55	0.378						
**Never-drinking**												
Liver cancer patients	370	274	120	130	72		0.241, 0.89 (0.73–1.08)	0.456, 1.12 (0.83–1.53)	0.138, 0.75 (0.51–1.10)	0.033, 0.67 (0.46–0.97)	0.950, 0.99 (0.75–1.32)	0.045, 0.71 (0.50–0.99)
Lung cancer patients	431	329	130	171	79		0.120, 0.86 (0.72–1.04)	0.605, 0.93 (0.69–1.24)	0.112, 0.74 (0.51–1.07)	0.219, 0.80 (0.56–1.14)	0.306, 0.87 (0.66–1.14)	0.129, 0.77 (0.56–1.08)
Normal controls	679	447	211	257	95	0.543						

1 Genotypic frequency of rs3787016 in normal controls was tested for departure from HWE using the χ^2^ test.

2 For each stratified factor, the *P* value and OR (95% CI) were calculated using two-sided χ^2^ test and logistic regression analysis. First row for ‘Liver cancer patients vs*.* Normal controls’, second row for ‘Lung cancer patients vs*.* Normal controls’.

### Results of meta-analysis

**Figure 2:**
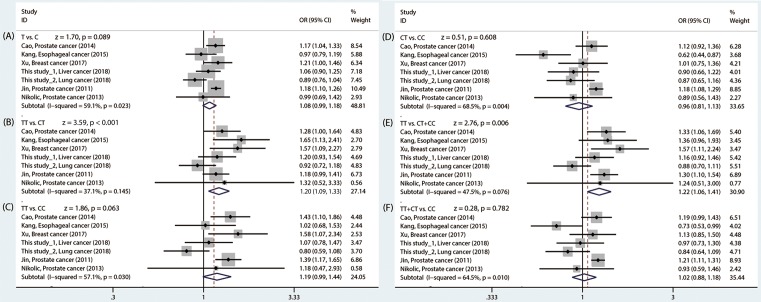
Forest plot for the association between POLR2E rs3787016 polymorphism and overall cancer risk. (**A**) T vs. C model, (**B**) TT vs. CT model, (**C**) TT vs. CC model, (**D**) CT vs. CC model, (**E**) TT vs. CT+CC model and (**F**) TT+CT vs. CC model.

**Figure 3:**
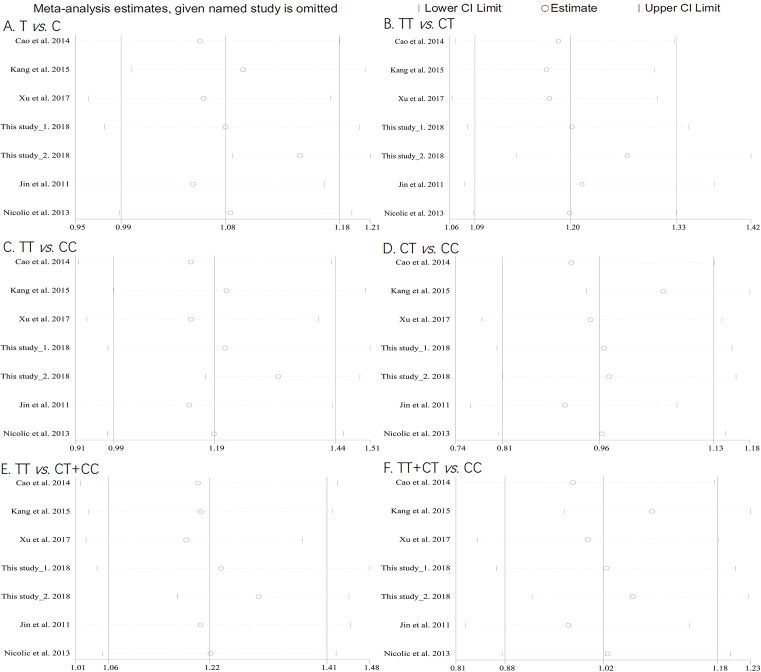
Sensitivity analysis for the association between POLR2E rs3787016 polymorphism and overall cancer risk. (**A**) T vs. C model, (**B**) TT vs. CT model, (**C**) TT vs. CC model, (**D**) CT vs. CC model, (**E**) TT vs. CT+CC model, and (**F**) TT+CT vs. CC model.

As shown in Supplementary Table S2, the NOS score of all articles are not <6, indicating that each included literature was a high-quality study. The main features of the five previous studies and current study were demonstrated in Table 4. All studies were consistent with HWE in normal controls (*P*>0.05). Similarly, the adjusted *P* value (<0.008, 0.05/6) using Bonferroni correction was applied. In Table 5, we observed that *POLR2E* rs3787016 was associated with cancer risk under the TT vs*.* CT model (*P*<1 × 10^−3^, OR = 1.20, 95% CI = 1.09–1.33) and TT vs. CT+TT model (*P*=0.006, OR = 1.22, 95% CI = 1.06–1.41), suggesting that the carriers with rs3787016 TT genotype had a significantly increased cancer risk compared with the CT/CC genotypes carriers (Figure 2). Further, we performed a sensitivity analysis to examine the stability of the pooled ORs with the effect of the individual studies. With removal of individual study results from the analysis for rs3787016, the pooled ORs remained significantly consistent (Figure 3). Next, stratified analysis according to ethnicity and cancer type was conducted. Interestingly, we found that rs3787016 was significantly associated with cancer risk in Caucasian population but not in Asian (Chinese) population. Moreover, the T allele and T variant genotypes of rs3787016 were associated with a significantly higher prostate cancer risk under the six genetic models (T vs. C, TT vs. CT, TT vs. CC, CT vs. CC, TT vs. CT+CC, and TT +CT vs. CC).

**Table 4:** Characteristics of the current and previous studies

References (author, year)	Ethnicity (Country)	Cancer type	Genotyping assay	Case, control (*n*)			HWE^1^
				Total	T/C	TT/CT/CC	
Cao et al. [9]	Asian (China)	Prostate cancer	PCR–RFLP	1015, 1032	891/1139, 826/1238	189/513/313, 151/524/357	0.180
Kang et al. [10]	Asian (China)	Esophageal cancer	PCR–RFLP	369, 370	329/409, 336/404	90/149/130, 71/194/105	0.268
Xu et al. [11]	Asian (China)	Breast Cancer	MassARRAY	439, 439	395/483, 354/524	93/209/137, 64/226/149	0.344
The present study	Asian (China)	Liver cancer	PCR–RFLP	480, 800	576/384, 936/664	188/200/92, 286/364/150	0.205
The present study	Asian (China)	Lung cancer	PCR–RFLP	550, 800	612/488, 936/664	181/250/119, 286/364/150	0.205
Jin et al. [7]	Caucasian (U.S.A.)	Prostate cancer	TaqMan assay	4196, 5007	2232/6160, 2354/7660	297/1638/2261, 277/1800/2930	0.997
Nikolic et al. [8]	Caucasian (Serbia)	Prostate cancer	TaqMan assay	261, 106	142/380, 58/154	21/100/140, 7/44/55	0.648

1 Genotypic frequency of rs3787016 in normal controls was tested for departure from HWE using the χ^2^ test.

**Table 5:** Meta-analysis of *POLR2E* rs3787016 polymorphism and cancer risk

Genetic model	Heterogeneity test			Summary OR (95% CI)	Hypothesis test		Number		
	*Q*	*P*	*I^2^*		*Z*	*P*	Case	Control	Studies
**rs3787016 and cancer risk**									
T vs*.* C	14.7	0.023	59%	1.08 (0.99–1.18)	1.70	0.089	14620	17108	7
TT vs*.* CT	9.54	0.145	37%	1.20 (1.09–1.33)	3.59	<1 × 10^−3^	4118	4658	7
TT vs. CC	14.0	0.030	57%	1.20 (0.99–1.44)	1.86	0.063	4251	5038	7
CT vs. CC	19.1	0.004	69%	0.96 (0.81–1.13)	0.51	0.608	6251	7412	7
TT vs. CT+CC	11.4	0.076	48%	1.22 (1.06–1.41)	2.76	0.006	7310	8554	7
TT+CT vs. CC	16.9	0.011	65%	1.02 (0.88–1.18)	0.28	0.782	7310	8554	7
**rs3787016 and cancer risk in Asian (Chinese)**									
T vs*.* C	10.1	0.039	60%	1.06 (0.94–1.19)	0.93	0.352	5706	6882	5
TT vs*.* CT	9.42	0.051	58%	1.26 (1.03–1.53)	2.25	0.024	2062	2530	5
TT vs. CC	11.2	0.025	64%	1.14 (0.89–1.46)	1.06	0.290	1532	1769	5
CT vs. CC	9.32	0.049	58%	0.90 (0.75–1.10)	1.02	0.308	2112	2583	5
TT vs. CT+CC	10.5	0.032	62%	1.21 (1.00–1.48)	1.91	0.056	2853	3441	5
TT+CT vs. CC	9.73	0.045	59%	0.97 (0.81–1.17)	0.30	0.764	2853	3441	5
**rs3787016 and cancer risk in Caucasian**									
T vs. C	0.86	0.353	0%	1.17 (1.10–1.25)	4.73	<1 × 10^−3^	8914	10226	2
TT vs. CT	0.06	0.813	0%	1.18 (1.00–1.41)	1.90	0.058	2056	2128	2
TT vs. CC	0.12	0.728	0%	1.38 (1.17–1.64)	3.73	<1 × 10^−3^	2719	3269	2
CT vs. CC	1.29	0.256	23%	1.17 (1.07–1.27)	3.59	<1 × 10^−3^	4139	4829	2
TT vs. CT+CC	0.01	0.914	0%	1.30 (1.10–1.53)	3.08	0.002	4457	5113	2
TT+CT vs. CC	1.22	0.270	18%	1.20 (1.10–1.30)	4.33	<1 × 10^−3^	4457	5113	2
**rs3787016 and prostate cancer risk**									
T vs. C	0.86	0.650	0%	1.17 (1.11–1.24)	5.36	<1 × 10^−3^	10944	12290	3
TT vs. CT	0.31	0.856	0%	1.21 (1.05–1.40)	2.68	0.007	2758	2803	3
TT vs. CC	0.16	0.921	0%	1.39 (1.21–1.61)	4.58	<1 × 10^−3^	3221	3777	3
CT vs. CC	1.47	0.480	0%	1.16 (1.07–1.25)	3.73	<1 × 10^−3^	4965	5710	3
TT vs. CT+CC	0.05	0.976	0%	1.31 (1.14–1.50)	3.91	<1 × 10^−3^	5472	6145	3
TT+CT vs*.* CC	1.23	0.542	0%	1.20 (1.11–1.29)	4.70	<1 × 10^−3^	5472	6145	3

## Discussion

LncRNAs play important roles in diverse human diseases including cancer, and abnormal expression of lncRNAs is a common feature of many human cancers [4,5]. Since SNPs can affect the gene expression and function [18], the lncRNAs polymorphisms have been widely studied to explore their associated with cancer risk [6].

The rs3787016 polymorphism, locates in an intron of *POLR2E* gene, was first reported in a genome-wide association study of prostate cancer [7]. Jin et al. [7] identified that *POLR2E* rs3787016 polymorphism was associated with prostate cancer susceptibility in Caucasian population. Subsequently, two replication studies on the possible association between rs3787016 and prostate cancer risk were conducted [8,9]. However, the significant association was found in Chinese population [9] but not in Serbian population [8]. Since a small number of subjects from Serbian population were included and different ethnic groups, we reasoned that the inconsistent results might be attributed to the differences in sample size and ancestral backgrounds.

Interestingly, Kang et al. [10] and Xu et al. [11] also revealed a significant association between rs3787016 with risk of esophageal cancer and breast cancer, which highlighted that *POLR2E* rs3787016 polymorphism might servers as a common genetic factor to affect individual susceptibility to cancer. To address this issue, for the first time, we here evaluated the association between rs3787016 and risk of liver and lung cancer. Although no significant association was found for rs3787016 and liver cancer or lung cancer risk, the further stratified analysis of rs3787016 according to age, gender, smoking status, and drinking status identified that rs3787016 exerted its effect on liver cancer risk particularly for over than 60 years individuals who drink. The interpretation of such finding might be as follow: aging and drinking might induce a variety of DNA damage or risk mutations and thus initiate liver carcinogenesis [19,20], and the effect of rs3787016 on liver cancer risk might be augmented by the factors of age and drinking. However, the interactions amongst rs3787016, aging and drinking in the etiology of liver cancer still needs to be investigated in further study.

Actually, Chu et al. [21] have performed a meta-analysis to evaluate the association between rs3787016 and cancer risk, which included the same studies [7–10]. However, we found that the data in study of Nikolic et al. [8] was wrongly extracted by Chu et al. Moreover, given the newly generated experiment data in current case–control study, we futher perform a rigorous and updated meta-analysis to determine the association of *POLR2E* rs3787016 polymorphism and cancer risk. We observed that rs3787016 was significantly associated with cancer risk in total population, and rs3787016 TT genotype contributed to a higher risk of cancer risk. However, the significant association remained in Caucasian population but not in Asian (Chinese) population, indicating that differences in genetic background may be a possible reflection of rs3787016 on cancer risk. In addition, the stratified analysis according to cancer type showed that the rs3787016 was associated with prostate cancer risk. However, further studies with larger sample size in different ethnic populations and in prostate cancer are warranted.

Admittedly, several limitations of the present study should be acknowledged. First, since a hospital-based case–control study was used, the potential for selection bias should be considered. Second, the underlying molecular mechanism for the contribution of rs937283 to cancer susceptibility remained unknown, which will be explored in future functional studies. Third, our current findings of this case–control study only involved Han Chinese population, thus further confirmatory studies in different ethnic groups are needed. Fourth, since the publication bias can be evaluated for meta-analysis with sufficient numbers of included studies (*n*>10), the assessment of publication bias was not performed through Begg’s funnel plot and Egger’s linear regression method [22]. Therefore, we could not eliminate the possibility of publication bias in the present study meta-analysis. Fifth, a high degree of heterogeneity was observed in the meta-analysis of rs3787016 and overall cancer risk in total population and Asian (Chinese) population. The variations of different cancer types, clinical characteristics, ethnicity, geographical location and so on were not fully considered. Sixth, due to the relatively small number of included studies, the subgroup analysis by cancer type only performed for prostate cancer, while for others, such as breast cancer and liver cancer, which should be investigated in the future. Finally, the *POLR2E* rs3787016 polymorphism may not be the causal loci, but may just be in linkage disequilibrium with the causal loci.

In summary, our results demonstrated that *POLR2E* rs3787016 polymorphism may be associated with the risk of liver cancer for over than 60 years Chinese individuals who drink. Moreover, the following meta-analysis revealed that *POLR2E* rs3787016 polymorphism may be associated with overall cancer risk and prostate cancer risk. Before these reported findings will contribute to clinical decision-making, additional studies with a larger sample size and in different ethnic populations are needed to confirm or further reinforce our present findings.

## Supporting information

**Supplementary Table 1:** The detailed search strategy

**Supplementary Table 2:** Quality assessment of the included studies according to the Newcastle-Ottawa Scale (NOS)

## Abbreviations

CI: confidence interval
HWE: Hardy–Weinberg equilibrium
LncRNA: long non-coding RNA
NOS: Newcastle-Ottawa Scale
OD: odds ratio
POLR2E: polymerase II polypeptide E
SNP: single nucleotide polymorphism
