# Supplementary material for: The association of POLR2E rs3787016 polymorphism and cancer risk: a Chinese case–control study and meta-analysis
Source: Biosci Rep. 2018 Nov 16;38(6):BSR20180853. doi: 10.1042/BSR20180853 (PMC6239260; doi:10.1042/BSR20180853)
Supplement: Supplementary file 1 [file bsr20180853_Supp1.pdf]

Supplementary Table 1. The detailed search strategy

(1) PUBMED database

Step 1: (#1) Number: 16

(((RNA Polymerase II Subunit E [MeSH Terms]) OR POLR2E) OR DNA-Directed RNA Polymerase II Subunit E) OR DNA-Directed RNA Polymerase Subunit RPABC1

Step 2: (#2) Number: 276827

((((Polymorphism [MeSH Terms]) OR Polymorphisms) OR mutation) OR mutations) OR SNP

Step 3: (#3) Number: 4126391

((((carcinoma [MeSH Terms]) OR cancer) OR adenocarcinoma) OR neoplasm) OR tumor

Step 4: (#1 And #2 And #3) Number: 7

(((((RNA Polymerase II Subunit E [MeSH Terms]) OR POLR2E) OR DNA-Directed RNA Polymerase II Subunit E) OR DNA-Directed RNA Polymerase Subunit RPABC1)) AND ((((((Polymorphism [MeSH Terms]) OR Polymorphisms) OR mutation) OR mutations) OR SNP)) AND ((((((carcinoma [MeSH Terms]) OR cancer) OR adenocarcinoma) OR neoplasm) OR tumor))

(2) WOS database

((TOPIC: (((RNA Polymerase II Subunit E OR POLR2E) OR DNA-Directed RNA Polymerase II Subunit E) OR DNA-Directed RNA Polymerase Subunit RPABC1) AND TOPIC: (((((polymorphism OR polymorphisms) OR mutation) OR mutations) OR SNP) OR Single Nucleotide Polymorphism)) AND TOPIC: ((((carcinoma OR cancer) OR adenocarcinoma) OR neoplasm) OR tumor))

Timespan: All years.

Search language=Auto

Number: 7

(3) EMBASE database

Step 1: (#1), Number: 41

'RNA Polymerase II Subunit E' OR 'POLR2E' OR 'DNA-Directed RNA Polymerase II Subunit E' OR 'DNA-Directed RNA Polymerase Subunit RPABC1'

Step 2: (#2), Number: 1151380

'polymorphism' OR 'polymorphisms' OR 'mutation' OR 'mutations' OR 'snp' OR 'single nucleotide polymorphism'

Step 3: (#3), Number: 3737805

'carcinoma' OR 'cancer' OR 'adenocarcinoma' OR 'neoplasm' OR 'tumor'

Step 4: (#1 And #2 And #3), Number: 8

'RNA Polymerase II Subunit E' OR 'POLR2E' OR 'DNA-Directed RNA Polymerase II Subunit E' OR 'DNA-Directed RNA Polymerase Subunit RPABC1' AND ('polymorphism' OR 'polymorphisms' OR 'mutation' OR 'mutations' OR 'snp' OR 'single nucleotide polymorphism') AND ('carcinoma' OR 'cancer' OR 'adenocarcinoma' OR 'neoplasm' OR 'tumor')

(4) WANFANG database
POLR2E * 癌症 + 肿瘤 * 基因多态性 * Date:-2018 * Date:1990-2018<br><br>**Number: 0**

(5) CNKI database
**Step 1: Number: 0**<br><br>主题=中英文扩展(POLR2E and 主题=中英文扩展(癌症 and 主题=中英文扩展(基因多态性 and (模糊匹配)<br><br>**Step 2: Number: 0**<br><br>主题=中英文扩展(TNF-α and 主题=中英文扩展(肿瘤 and 主题=中英文扩展(基因多态性 and (模糊匹配)

(6) SCOPUS database
(TITLE-ABS-KEY ("RNA Polymerase II Subunit E" OR "POLR2E" OR "DNA-Directed RNA Polymerase II Subunit E" OR "DNA-Directed RNA Polymerase Subunit RPABC1") AND TITLE-ABS-KEY ("polymorphism" OR "polymorphisms" OR "mutation" OR "mutations" OR "SNP" OR "Single Nucleotide Polymorphism") AND TITLE-ABS-KEY ("carcinoma" OR "cancer" OR "adenocarcinoma" OR "neoplasm" OR "tumor"))<br><br>**Number: 8**

Supplementary Table 2. Quality assessment of the included studies according to the Newcastle-Ottawa Scale (NOS)

Author (Ref)	Year	Case		Control		Comparability		Exposure				NOS score
		Definition	Represent-ativeness	Selection	Definition	Important factors	Other factors	Secure record	Blind	Method	Non-response rate	
**Jin et al.**	2011	★	★	★	★	★	★	★	☆	★	★	**9**
**Nikolic et al.**	2013	☆	★	★	★	★	★	☆	☆	★	☆	**6**
**Cao et al.**	2014	★	★	★	★	★	★	☆	☆	★	★	**8**
**Kang et al.**	2015	★	★	★	★	☆	★	☆	☆	★	☆	**6**
**Xu et al.**	2017	★	★	★	★	★	☆	☆	☆	★	☆	**6**
**This study_1**	2018	☆	★	★	★	★	★	☆	☆	★	★	**7**
**This study_2**	2018	☆	★	★	★	★	★	☆	☆	★	★	**7**

★, score value=1; ☆, score value=0; The specific item information is available from http://www.ohri.ca/programs/clinical_epidemiology/oxford.asp.
